# Mitonuclear interactions may contribute to fitness of fungal hybrids

**DOI:** 10.1038/s41598-018-19922-w

**Published:** 2018-01-26

**Authors:** Luana Giordano, Fabiano Sillo, Matteo Garbelotto, Paolo Gonthier

**Affiliations:** 1 University of Torino, Department of Agricultural, Forest and Food Sciences (DISAFA), Largo Paolo Braccini 2, I-10095 Grugliasco (TO), Italy; 2 Centre of Competence for the Innovation in the Agro-Environmental Field (AGROINNOVA), University of Torino, Largo Paolo Braccini 2, I-10095 Grugliasco (TO), Italy; 3 University of California, Berkeley, Department of Environmental Science, Policy and Management, Forest Pathology and Mycology Laboratory, 54 Mulford Hall, 94720 Berkeley, California USA

## Abstract

Hybridization between species is being recognized as a major force in the rapid adaptive evolution of fungal plant pathogens. The first stages of interspecific hybridization necessarily involve nuclear-mitochondrial chimeras. In their 2001 publication, Olson and Stenlid reported that mitochondria control the virulence of first generation hybrids between the North American fungal pathogen *Heterobasidion irregulare* and its congeneric *H*. *occidentale*. By assessing saprobic ability and gene expression of *H*. *irregulare* × *H*. *annosum sensu stricto* hybrids and of their parental genotypes, we demonstrate that mitochondria also influence saprobic growth of hybrids. Moreover, gene expression data suggest that fungal fitness is modulated by an intimate interplay between nuclear genes and mitochondrial type, and is dependent on the specific mitonuclear combination.

## Introduction

**Figure 1:**
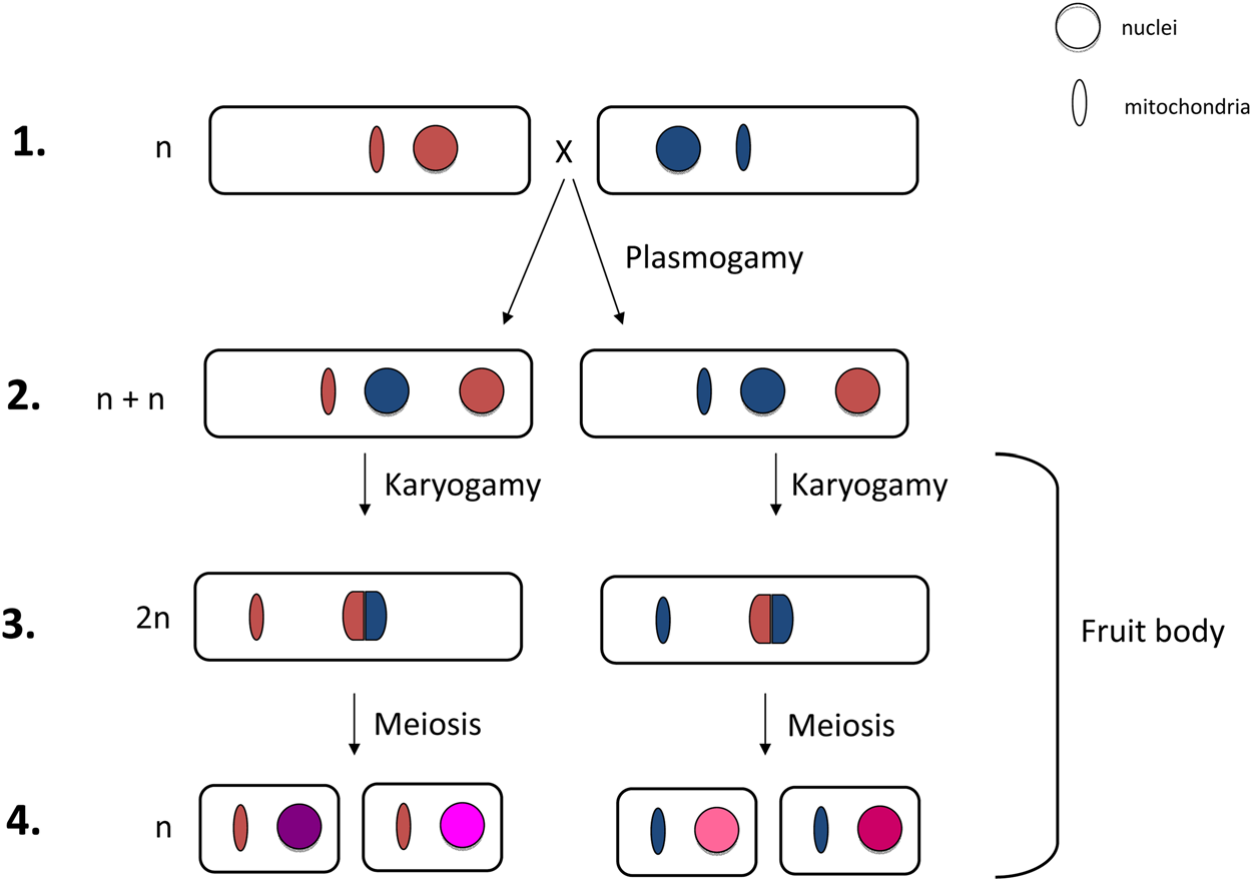
Schematic representation of hybridization in Basidiomycetes. (**1**) Haploid (n) mycelia originated from meiospores carrying compatible mating types of two interfertile species met and through hyphal anastomosis they fuse. Plasmogamy occurs and nuclei can migrate. (**2**) Resulting F1 hybrids are dikaryons/heterokaryons (n + n), carrying both parental nuclei but only one mitochondrial type. (**3**) Heterokaryotic (n + n) mycelium is the main vegetative and growth phase. When mature, fruit bodies will be produced where karyogamy occurs, producing a diploid cell (2n). (**4**) After karyogamy, meiosis generates new haploid meiospores (n) that contain an admixture of the parental genomes (F2 hybrids).

Fungal hybridization is a process recently acknowledged to occur in nature more frequently than originally thought^1–3^. In basidiomycetous fungi, first generation (F1) hybrids are generated through plasmogamy of two haploid mycelia (n) belonging to different interfertile species carrying compatible mating types. As in all Basidiomycetes, karyogamy is delayed, and the extensive life phase in between plasmogamy and karyogamy represents the main vegetative or growth phase of hybrid mycelia. Such phase is characterized by the presence of nuclear haploid genomes from both parental species and is known as heterokaryotic (n + n). Once the heterokaryotic mycelium is well established and has sufficiently colonized its substrate, fruit bodies are produced in which karyogamy and meiosis occur resulting in the production of haploid meiospores, responsible for spread of the fungus^2^ (Fig. 1). In the majority of Basidiomycetes, including the genus *Heterobasidion*, nuclei migrate between mating individuals but mitochondria do not^4^. As a result, mitochondria are inherited from only one of the parental species^4^ (Fig. 1). As in plants and animals, the uniparental inheritance of mitochondria in fungi is a genetically regulated process, often leading to the selective removal of one of the two parental mitochondria in natural populations^4,5^.

Hybrid breakdown has been observed in several studies on F1 hybrids between species that were once living in allopatry^6^. Fitness reduction in hybrids is being recognized as the consequence of incompatibility between mitochondrial and nuclear alleles, plausibly according to the classical biological model of evolution of genetic incompatibility known as the Bateson-Dobzhansky-Muller incompatibility (BDMI) model^7^. For example, mitonuclear interactions in yeasts have been reported to affect growth^8^, to reinforce reproductive isolation^9,10^, and to result in phenotypes that are more sensitive to environmental extremes compared to their parental strains^11,12^. Nonetheless, the current knowledge on the role and the effect of mitonuclear interactions in fungal hybrids is still limited^13^.

Olson and Stenlid^14^ generated interspecific F1 heterokaryotic hybrids between the North American pine pathogen *Heterobasidion irregulare* and the North American non-pine pathogen *H*. *occidentale* and performed inoculation studies on pine germlings to show that hybrids with the *H*. *irregulare* mitochondria were more virulent than hybrids with the *H*. *occidentale* mitochondria. That report was one of the first to highlight the importance of mitochondria in the infection process of a host by a pathogen, and to provide timely evidence on the fitness of fungal hybrids. Since both heteroplasmy of distinct mitochondria in the same individual and mitochondrial recombination occur rarely in fungi^15^, reduced fitness cannot be ascribed to the co-occurrence of different mitochondria in the same cell or to the emergence of new mitochondrial genomes. Rather, fitness may be the result of conflicting, albeit specific, interactions between nuclear and mitochondrial genomes. Notwithstanding that *H*. *irregulare* × *H*. *occidentale* F1 hybrids appear to be extremely rare in nature^16^, the experimental design of Olson and Stenlid was adequate to uncover a likely mitochondrial control of virulence, although without providing any explanation of the mechanisms involved.

It has recently been reported that *H*. *irregulare* is massively hybridizing with the European species *H. annosum sensu stricto* (*s.s.*) across multiple sites in Central Italy^17^. Sympatry between the two species can be dated to 1944, when the North American species was introduced in Europe. Interestingly, *H. irregulare* is clearly dominant and outcompetes the native *H. annosum s.s.*, in spite of its relatively recent history in Italy^18^. Its dominance in Central Italy is associated not with higher pathogenicity on the main Italian host, *Pinus pinea*, but rather with its higher saprobic and sporulation potential, i.e. its greater ability to grow on wood substrates and to produce fruit bodies^19,20^. These two traits ensure high establishment rates in forest stands because several *Heterobasidion annosum sensu lato* (*s.l.*) species become established through the infection of stumps by airborne spores, followed by saprobic colonization of infected stumps and stumps’ roots by vegetative mycelium^16^. A significant proportion of genotypes across the entire invasive range of *H. irregulare* are actually admixtures of *H. irregulare* and *H. annosum s.s.* nuclear genomes, but the overwhelming majority of them are characterized by a *H. irregulare* mitochondrial genome^17^. This observation prompted us to confirm that fitness of admixed genotypes may be correlated with the mitochondrial genome.

## Results and Discussion

**Figure 2:**
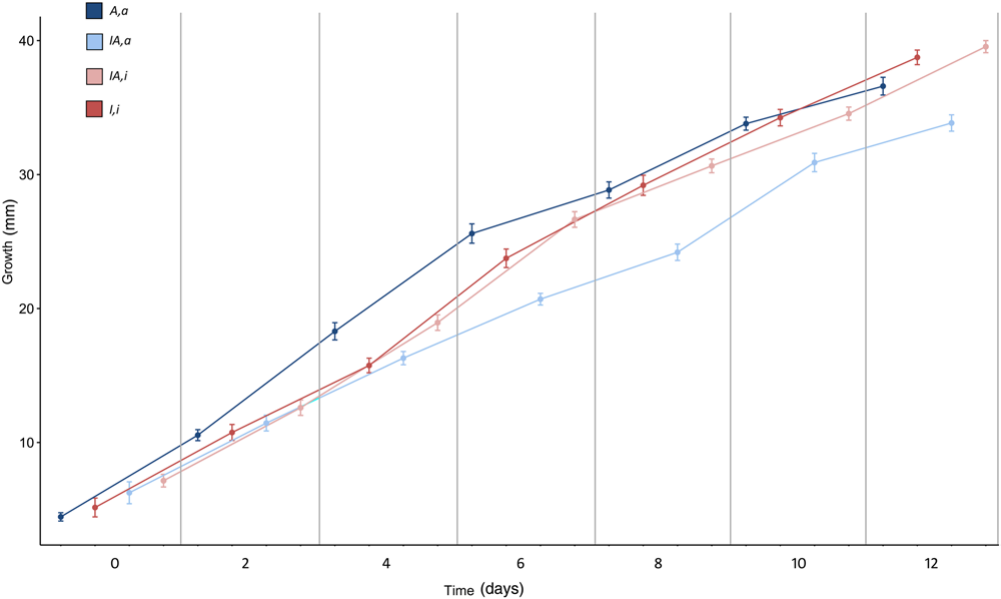
Growth curves of the four genotypes during saprobic assay. Each line represents the average of ten replicates. Error bars indicate ± Standard Deviation (SD). Points of x-axis were offset to improve the readability.

Given that saprobic growth is a key trait driving the invasion by *H*. *irregulare*, fitness of *H*. *irregulare* × *H*. *annosum s*.*s*. F1 hybrids was evaluated by measuring their saprobic growth potential on pine wood. Four genotypes were employed, namely a pure haploid *H*. *irregulare* genotype with a *H*. *irregulare* mitochondrion (*I*,*i*), a pure haploid *H*. *annosum s*.*s*. genotype with a *H*. *annosum s*.*s*. mitochondrion (*A*,*a*), the *H*. *irregulare* −* H*. *annosum s*.*s*. heterokaryotic (n + n) hybrid with a *H*. *irregulare* mitochondrion (*IA*,*i*), and the same heterokaryotic hybrid with the *H*. *annosum s*.*s*. mitochondrion (*IA*,*a*). Both hybrids were generated by mating the same two pure haploid genotypes above. Although mycelial growth of *I*,*i* and *A*,*a* was similar (38.75 *vs*. 36.60 mm; p = 0.108 with Bonferroni correction), unexpectedly, growth of *IA*,*i* differed significantly from that of *IA*,*a* (39.55 *vs*. 33.85 mm; p < 0.001) (Fig. 2 and Supplementary Table 1). In fact, while the hybrid *IA*,*i* had an average mycelial growth indistinguishable from that of the pure *H*. *irregulare* genotype *I*,*i* (39.55 *vs*. 38.75 mm; p = 1.000), the hybrid *IA*,*a* had a growth significantly lower than the pure *H*. *annosum s*.*s*. genotype *A*,*a* (33.85 *vs*. 36.60 mm; p = 0.036; Fig. 2 and Supplementary Table 1). These results confirmed that fitness of hybrids is associated with the mitochondrial genome, and indicated that a depression of fitness is to be expected when the *H*. *annosum s*.*s*. mitochondrion is combined with the *H*. *irregulare* nucleus. Conversely, the opposite is not true, as evidenced by the high fitness recorded for the *IA*,*i* hybrid.

**Figure 3:**
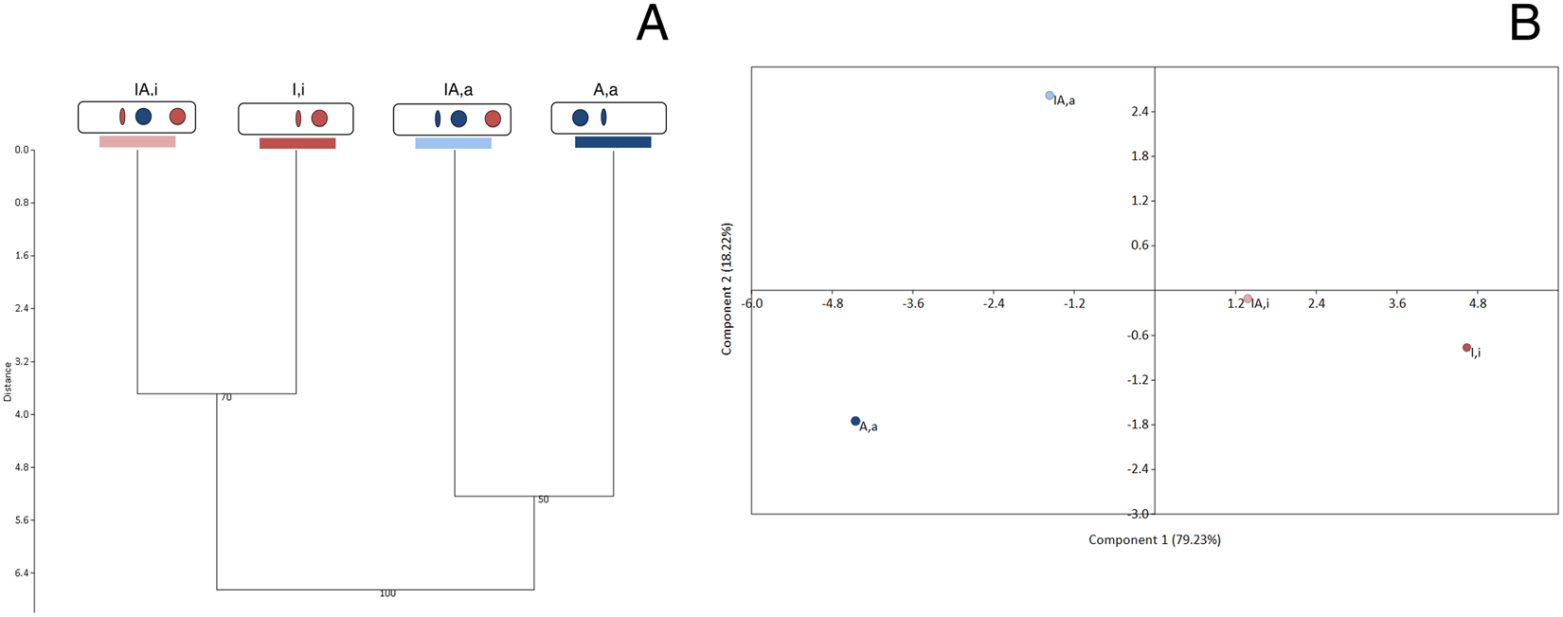
Results of hierarchical clustering and Principal Component Analysis (PCA) on gene expression data. (**A**) Paired UPGMA tree (Euclidean distance; 1000 bootstrap iterations). Bootstrap values >50 are shown. (**B**) PCA. Percentages of variance explained for the two axis are reported.

Expression levels for each of 9 nuclear genes, for which gene products were reported to be putatively involved in wood and host colonization in previous studies^21–23^, were measured and compared among all four genotypes, between each hybrid and the pure genotype carrying the same mitochondrial genome, and between the two hybrids. Hierarchical clustering analysis on gene expression data (Fig. 3) showed that the overall pattern of gene expression during saprobic growth was strongly correlated with mitochondrial type, rather than with nuclear composition. Consequently, overall gene expression pattern of the *IA*,*a* hybrid clustered with that of the *A*,*a* genotype, while that of *IA*,*i* clustered with that of *I*,*i*. However, Principal Component Analysis (PCA) on the same dataset showed that *I*,*i* and *IA*,*i* were closest in terms of overall gene expression, while *A*,*a* and *IA*,*a* were much less similar to one another (Fig. 3).

**Figure 4:**
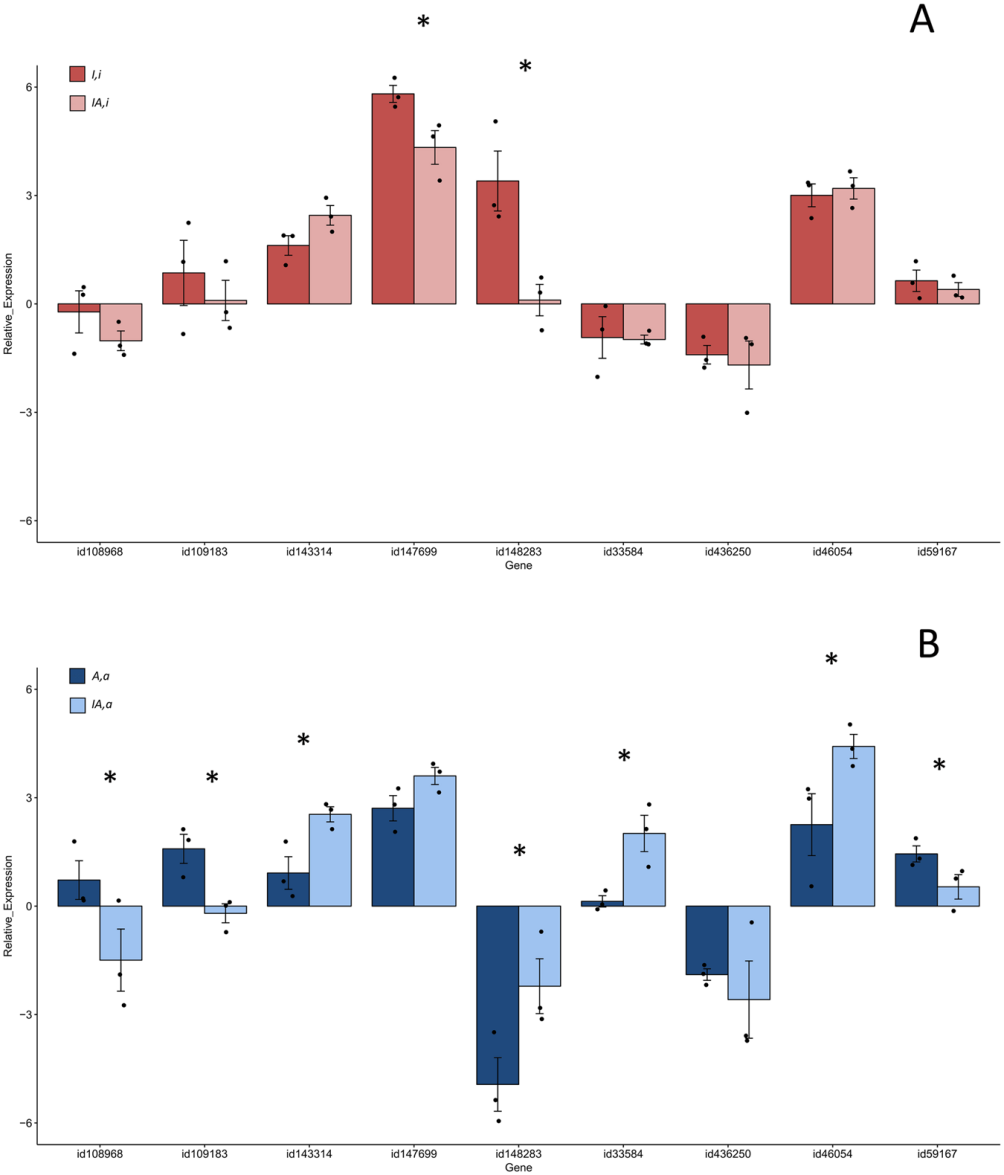
Relative gene expression of the four genotypes during the saprobic assay. (**A**) Barplot of relative gene expression of *I,i* and *IA,i*. (**B**) Barplot of relative gene expression of *A,a* and *IA,a*. Bars represent the average relative expression normalized to the internal control (reference gene) for each genotype (∆CTs). Black dots represent relative expression values for each replicate. Error bars indicate ± Standard Error (SE). Asterisks indicate significant differences (p < 0.05) as inferred by the Pair Wise Fixed Reallocation Randomisation Test^©^.

In order to investigate differences in interactions between the *H*. *annosum s*.*s*. mitochondrion (*a*) and the *H*. *irregulare* nucleus (*I*), as opposed to those between the *H*. *irregulare* mitochondrion (*i*) and the *H*. *annosum s*.*s*. nucleus (*A*), we compared gene expression of the pure genotype *A*,*a* with that of the hybrid *IA*,*a*, and gene expression of *I*,*i* with that of *IA*,*i*. Results showed that 7 out of 9 genes were differentially expressed in the *IA*,*a* hybrid compared to the pure *A*,*a* genotype (Fig. 4 and Supplementary Table 2), while only 2 genes were differentially expressed in the *IA*,*i* hybrid compared to the pure *I*,*i* genotype (Fig. 4 and Supplementary Table 3). We hypothesize that the significant changes in gene expression levels (both under- and over-expression) detected in hybrids with the *H*. *annosum s*.*s*. mitochondrion may be the result of a costly lack of compatibility between the mitochondrion of that species and the *H*. *irregulare* nucleus. Therefore, the negative effect on fitness may be the result of a significantly imbalanced presence of transcripts when compared to transcriptomic profile of the parental genotype.

In contrast, the hybrid carrying the *H*. *irregulare* mitochondrion more closely matches the pure *H*. *irregulare* genotype in gene expression.

Finally, we also compared gene expression data of *IA*,*a* versus *IA*,*i*. Interestingly, 3 out of 9 genes belonging to three different categories, i.e. a gene putatively encoding an hydrophobin-like protein (*46054*), a gene putatively encoding a fatty acid desaturase with oxidoreductase activity (*148283*), and one putatively encoding a mitochondrial carrier (*33584*), were significantly (p < 0.05) differentially regulated (Supplementary Table 4). This result suggested that different mitochondrial types may affect different gene categories in F1 hybrids.

It should be noted that in order to compare gene expression of all four genotypes, we opted to focus on transcripts of regions that are conserved between the two *Heterobasidion* species for the 9 selected genes. Thus, the role played by each of the two species in the generation of the analyzed transcriptome cannot be determined. However, while we cannot exclude that gene expression of heterokaryotic F1 hybrids *IA*,*i* and *IA*,*a* may be biased by different nuclear ratios of the two parental species as observed in other *Heterobasidion* species^24,25^, there is yet no evidence that the mitochondrial genome may affect nuclear ratios^24^. Hence, we conclude that differences in gene expression may be due to differential interactions between nuclei and the mitochondria.

Our results showed that mitochondria influence saprobic growth in *H*. *irregulare* × *H*. *annosum s*.*s*. hybrids, and that expression of a set of nuclear genes involved in wood and host colonization varies in association with different mitonuclear combinations. It should be noted that nuclear genomes of *H*. *irregulare* and *H*. *annosum* *s.s.* have been reported to be very similar, i.e. 98% of interspecific nucleotide homology^23^. Although no information on divergence between mitochondrial genomes is available, it could be assumed that the mitochondrial genomes may be similar as well. Based on this assumption, it is unlikely that mitochondrial genomes themselves may be responsible for fitness reduction in hybrids, though we cannot exclude that they may encode factors directly involved in saprobic growth. Our results strongly support the hypothesis that the interplay between mitochondria and nuclei may be crucial to determine the phenotype of hybrids. Moreover, the asymmetry of the results in both phenotypic and transcriptomic experiments indicates that it is the particular mitonuclear combination that determines the results and not just a general mismatch between mitochondrion and nucleus. Such finding supports previous observations by Olson and Stenlid^14^.

We acknowledge that our findings, although convincing, will require to be corroborated by further studies because they are based on a single combination of two genotypes of the two species, and on gene expression analysis of a selected set of representative genes. Nonetheless, this work is one of the first to highlight a possible and plausible role of mitonuclear interactions in determining the fitness of fungal hybrids in broader terms than previously done. This study, however, not only expands the notion of the association between fitness and mitochondria introduced by Olson and Stenlid using virulence, but also provides an explanation of the reason why hybrids carrying the *H*. *annosum s*.*s*. mitochondrion may be at a significant disadvantage in Italian admixed populations.

Previous phylogenetic studies suggested that *H*. *annosum s*.*s*. is more similar to the common ancestor of the two species than *H*. *irregulare*^26^. Furthermore, it has been suggested that, in the absence of recombination, mitochondrial evolution may predate nuclear evolution^27^. In North America, the newly evolved *H*. *irregulare* mitochondrion may have interacted with a nuclear genome resembling to *H*. *annosum s*.*s*. Conversely, in Eurasia, the *H*. *annosum s*.*s*. mitochondrion never interacted with the allopatrically evolved *H*. *irregulare* nuclear genome, until the recent introduction of *H*. *irregulare* in Italy^17^. As a result, natural selection may have already ‘tested’ and selected fit genotypes with *H*. *irregulare* mitochondria and nuclear genomes similar to *H*. *annosum s*.*s*., but the same is not true with genotypes bearing *H*. *annosum s*.*s*. mitochondria and *H*. *irregulare* nuclear genomes. This scenario is consistent with the BDMI model of mitonuclear interactions proposed by Sloan and colleagues^28^.

In summary, this study shows that hybrids carrying a nucleus of *H*. *irregulare* and mitochondria of *H*. *annosum s*.*s*. have a reduced saprobic ability, thus limiting their establishment in the environment. Highly differential genic expression, i.e. ‘expression bias’^29^, in *IA*,*a* compared to the pure parent with the same mitochondrion may be regarded as the result of an energetic cost for F1 hybrids carrying the *H*. *annosum s*.*s*. mitochondria. This hypothesis fits our results and the recently proposed model of speciation based on mitonuclear epistatic interactions^28^.

## Methods

### Generation of artificial hybrids and saprobic assay

Hybrids F1 were obtained by mating *in vitro* one pure *H. irregulare* and one pure *H. annosum s.s.* genotype. In detail, mycelial plugs 6 mm in diameter were removed with a cork borer from actively growing cultures (7 days old) and were paired by placing the plugs about 10 mm apart and 30 mm from the edge of a 90-mm Petri dish containing Malt Extract Agar (2% w/v). Petri dishes were incubated at room temperature (RT) (25 °C) under light. To verify that mating between genotypes occurred, presence of clamp connections in the contact zone was assessed under a dissecting microscope (20X magnification). In addition, molecular analysis as previously described^18^ was also performed to confirm the presence of either *H. irregulare* or *H. annosum s.s.* mitochondria in heterokaryotic hybrids. Two hybrids with the same nuclear genome but with mitochondrial genomes of either species were obtained by transferring pieces of mycelia taken from sectors behind the inocula into Malt Extract Agar in Petri dishes as previously described^30,31^. All fungal genotypes were deposited at the *Mycotheca Universitatis Taurinensis* (MUT) (see Supplementary Table 5). Saprobic assays were performed in 90-mm Petri dishes filled with *P. pinea* sawdust and water agar (15 g of *P. pinea* sawdust and 12 g of agar *per* liter). Parents (*I,i* and *A,a*) and F1 hybrids (*IA,i* and *IA,a*) were inoculated on the center of 90-mm Petri dishes. Ten replicates for each pair were used and Petri dishes were incubated at RT (25 °C) at dark, to limit any possible bias due to light conditions which can affect fungal phenotype^32^. Mycelial growth expressed in mm of colonization (two measures of radial growth for each replicate) was measured every 48 hours for 15 days, and significant (p < 0.05) differences were assessed through Kruskal Wallis and Mann-Whitney U tests at the end of the experiment.

### Gene expression analyses

Nine primer pairs were designed by using Primer3Plus (http://www.bioinformatics.nl/cgi-bin/primer3plus/primer3plus.cgi/) to target genes chosen for their role in wood and host colonization processes. In particular, all selected genes are reported to be expressed during growth in lignin and cellulose-based media, in wood and in infected bark, according to microarray data^21^. Five out of 9 (*147699*, *148283*, *143314*, *108968*, and *59167*) putatively encode for catalytic proteins with oxidoreductase activity; one (*33584*) for a mitochondrial substrate carrier; one (*46054*) for an hydrophobin-like protein; one (*109183*) for a factor putatively involved in the secondary metabolite pathway; one (*436250*) for a transcription factor. The genes *147699*, *148283*, *143314* and *109183* were also selected because their transcripts were detected in large scale transcriptomic analysis both in *H. irregulare* and *H. annosum s.s.*^21,22^. In addition, all selected genes showed signs of positive selection (high dN/dS ratio) according to a whole-genome analysis of several genotypes of the two species^23^. Full-length sequences of candidate genes were obtained from the available *H. irregulare* genome (http://genome.jgi-psf.org/Hetan2/Hetan2.home.html). Primers were designed on conserved genic regions between *H. irregulare* and *H. annosum s.s.* on the basis of whole-genome alignments previously performed^23^. RNA was extracted from mycelia of three biological replicates *per* genotype at the end of the saprobic assay (15^th^ day). RNA extraction was carried out using Spectrum™ Plant Total RNA Kit (Sigma-Aldrich, Saint Louis, Missouri, USA) following the manufacturer’s protocol. RNA was cleaned of DNA using RQ1 RNase-Free DNase (Promega Corp., Madison, WI, USA), and then quantified using a NanoDrop (Thermo Scientific, Hudson, NH, USA). The absence of genomic DNA was verified through PCR with primers for gene *46054*. The absence of signal after PCR amplification without retrotranscription was regarded as absence of DNA. Four hundred ng of total RNA were used for each sample to synthesize the cDNA, according to the SuperScript II Reverse Transcriptase (Invitrogen, Carlsbad, CA, USA) protocol. Quantitative RT-PCRs (RT-qPCRs) were carried out with the Connect™ Real-Time PCR Detection System (Bio-Rad Laboratories, Hercules, CA, USA). Each RT-qPCR reaction was carried out on a total volume of 20 μl, containing 2 μl diluted cDNA (dilution 1:3), 15 μl SsoAdvanced™ Universal SYBR^®^ Green Supermix (Bio-Rad Laboratories) and 1.5 μl of each primer (3 μM), using a 96-well plate. Primer sequences with their optimal annealing temperature are listed in Supplementary Table 6. The following PCR program, which includes the calculation of the melting curve, was used: 95 °C for 30 seconds, 40 cycles of 95 °C for 10 seconds, the optimal annealing temperature for 30 seconds, ramp from 65 °C to 93 °C with a temperature increment of 0.5 °C and a read plate every 2 seconds. All reactions were performed for three biological and three technical replicates. The baseline range and Ct values were automatically calculated using the Bio-Rad CFX Manager software. The candidate gene expression was normalized to that of the reference gene by subtracting the Ct value of the reference gene *Tryp Metab* (Protein ID: 43087)^33^ from the Ct value of the candidate gene with efficiency correction, from equation 2^−ΔΔCT^, where ΔΔCt represents the ΔCt sample − ΔCt control^34^. The gene *Tryp Metab* was selected as reference given its high expression stability in different tissues and samples based on previous experiments^33^. Raw Ct values for each replicate were reported in Supplementary Table 7.

Significant differences in gene expression (p < 0.05) were assessed through the Pair Wise Fixed Reallocation Randomisation Test^©^^35^ performed by using the Relative Expression Software Tool REST^©^ 2009 v. 2.0.13. Hierarchical clustering (Paired UPGMA tree) based on 1000 bootstrap replications and Principal Component Analysis (PCA) were performed on gene expression data (2^−∆CTs^) by using the statistical software PAST v. 3.16^36^.

### Data availability

All data generated or analysed during this study are included in this article (and its Supplementary Information files).

## Electronic supplementary material

Supplementary Information
