# Supplementary material for: Mitonuclear interactions may contribute to fitness of fungal hybrids
Source: Sci Rep. 2018 Jan 26;8:1706. doi: 10.1038/s41598-018-19922-w (PMC5786003; doi:10.1038/s41598-018-19922-w)
Supplement: Supplementary file 1 — Supplementary Information [file 41598_2018_19922_MOESM1_ESM.pdf]

## Supplementary Information for

# Mitonuclear interactions may contribute to fitness of fungal hybrids

**Luana Giordano[1,2], Fabiano Sillo[1*], Matteo Garbelotto[3], Paolo Gonthier[1]**

[1]University of Torino, Department of Agricultural, Forest and Food Sciences (DISAFA), Largo Paolo Braccini 2, I-10095 Grugliasco (TO), Italy

[2]Centre of Competence for the Innovation in the Agro-Environmental Field (AGROINNOVA), University of Torino, Largo Paolo Braccini 2, I-10095 Grugliasco (TO), Italy

[3]University of California, Berkeley, Department of Environmental Science, Policy and Management, Forest Pathology and Mycology Laboratory, 54 Mulford Hall, 94720 Berkeley, California, USA

[*]Corresponding author: **Fabiano Sillo -** fabiano.sillo@unito.it

**Supplementary tables**

Table S1……………………………………………………………………………………2

Table S2……………………………………………………………………………………3

Table S3……………………………………………………………………………………3

Table S4…………………………………………………………………………….. …..3

Table S5…………………………………………………………………………….. …..4

Table S6…………………………………………………………………………….. …..4

Table S7…………………………………………………………………………….. …..5

**Table S1**. Radial growth (average measure in mm ± SD) of the four different genotypes (ten replicate per genotype).

| Genotype | Replicate # | 3 days | 5 days | 7 days | 9 days | 11 days | 13 days | 15 days |
|---|---|---|---|---|---|---|---|---|
| *A,a* | 1 | 5.5±2.12 | 12.5±0.71 | 18.5±0.71 | 26.5±2.12 | 29.5±2.12 | 34.5±0.71 | 40±0 |
| *A,a* | 2 | 3.5±0.71 | 9.5±0.71 | 17±1.41 | 23.5±2.12 | 27±1.41 | 32.5±0.71 | 35±0 |
| *A,a* | 3 | 2.5±0.71 | 8.5±0.71 | 17±0 | 25.5±0.71 | 29±1.41 | 32.5±0.71 | 34±1.41 |
| *A,a* | 4 | 4.5±0.71 | 10.5±0.71 | 19.5±0.71 | 27±0 | 30±0 | 35±1.41 | 37±0 |
| *A,a* | 5 | 4±0 | 9.5±0.71 | 17±0 | 24.5±0.71 | 27.5±0.71 | 33.5±0.71 | 36±0 |
| *A,a* | 6 | 5±1.41 | 11.5±0.71 | 20±1.41 | 28.5±0.71 | 31.5±0.71 | 35.5±0.71 | 37.5±0.71 |
| *A,a* | 7 | 6±2.83 | 12±1.41 | 22.5±0.71 | 28.5±0.71 | 31.5±0.71 | 36±0 | 40±0 |
| *A,a* | 8 | 4.5±0.71 | 9.5±0.71 | 17±0 | 25±1.41 | 28±1.41 | 34±1.41 | 36.5±0.71 |
| *A,a* | 9 | 4.5±0.71 | 11.5±0.71 | 19±1.41 | 26±1.41 | 29±0 | 33.5±0.71 | 35.5±0.71 |
| *A,a* | 10 | 4.5±0.71 | 10.5±0.71 | 15.5±0.71 | 21±1.41 | 25.5±2.12 | 31±2.83 | 34.5±2.12 |
| *I,i* | 1 | 6.5±0.71 | 12±0 | 16±1.41 | 24±1.41 | 31±1.41 | 35.5±0.71 | 40±0 |
| *I,i* | 2 | 6±0 | 11.5±0.71 | 16.5±0.71 | 25.5±2.12 | 31±1.41 | 36.5±0.71 | 40±0 |
| *I,i* | 3 | 8±0 | 14±0 | 18.5±0.71 | 26.5±0.71 | 32±1.41 | 36.5±2.12 | 40±0 |
| *I,i* | 4 | 6±0 | 12±0 | 17±0 | 25.5±0.71 | 31.5±2.12 | 36.5±2.12 | 40±0 |
| *I,i* | 5 | 5±0 | 10±0 | 15±0 | 21.5±0.71 | 27.5±0.71 | 32.5±2.12 | 36±0 |
| *I,i* | 6 | 7±1.41 | 12±0 | 17.5±0.71 | 26±1.41 | 30.5±2.12 | 34±1.41 | 40±0 |
| *I,i* | 7 | 4.5±0.71 | 9.5±0.71 | 15.5±0.71 | 23.5±0.71 | 29±0 | 34±0 | 40±0 |
| *I,i* | 8 | 5±0 | 10±0 | 15±1.41 | 23.5±0.71 | 28±0 | 33.5±0.71 | 38±0 |
| *I,i* | 9 | 3.5±0.71 | 9±1.41 | 13.5±0.71 | 21.5±0.71 | 26.5±0.71 | 32.5±0.71 | 37.5±0.71 |
| *I,i* | 10 | 0±0 | 7.5±0.71 | 13±0 | 20±0 | 25±0 | 31±1.41 | 36±1.41 |
| *IA,a* | 1 | 7±1.41 | 13±1.41 | 17.5±2.12 | 21±1.41 | 24±0 | 30±1.41 | 32.5±2.12 |
| *IA,a* | 2 | 8±0 | 13±0 | 17±0 | 20±0 | 23±0 | 29±0 | 33±0 |
| *IA,a* | 3 | 8±0 | 12±0 | 17±1.41 | 21±1.41 | 28±1.41 | 35±0 | 37±0 |
| *IA,a* | 4 | 0±0 | 8.5±0.71 | 14.5±0.71 | 19.5±0.71 | 23.5±2.12 | 32±1.41 | 34.5±2.12 |
| *IA,a* | 5 | 7.5±0.71 | 13.5±0.71 | 16±0 | 21.5±0.71 | 26±1.41 | 32.5±0.71 | 34.5±0.71 |
| *IA,a* | 6 | 6±0 | 10±1.41 | 14.5±0.71 | 19±1.41 | 22±2.83 | 30.5±0.71 | 33.5±0.71 |
| *IA,a* | 7 | 8±0 | 13.5±0.71 | 19±0 | 22.5±0.71 | 24.5±0.71 | 32.5±0.71 | 35.5±0.71 |
| *IA,a* | 8 | 3.5±2.12 | 9±1.41 | 14±0 | 18.5±0.71 | 21.5±0.71 | 28.5±2.12 | 32±2.83 |
| *IA,a* | 9 | 7.5±0.71 | 10.5±0.71 | 16.5±2.12 | 22±1.41 | 24±1.41 | 31±1.41 | 35.5±2.12 |
| *IA,a* | 10 | 7±0 | 11.5±0.71 | 17±0 | 22±0 | 25.5±0.71 | 28±0 | 30.5±0.71 |
| *IA,i* | 1 | 6.5±0.71 | 11±1.41 | 17.5±0.71 | 25.5±2.12 | 31±1.41 | 36±1.41 | 40±0 |
| *IA,i* | 2 | 6±0 | 11.5±0.71 | 17.5±2.12 | 25.5±0.71 | 29.5±0.71 | 33.5±0.71 | 40±0 |
| *IA,i* | 3 | 7.5±0.71 | 13±0 | 20±0 | 26.5±2.12 | 31±2.83 | 35±4.24 | 40±0 |
| *IA,i* | 4 | 9.5±0.71 | 16.5±2.12 | 21±1.41 | 28±0 | 31±0 | 34.5±2.12 | 40±0 |
| *IA,i* | 5 | 7±0 | 12±0 | 17±1.41 | 27.5±0.71 | 32±0 | 35.5±0.71 | 40±0 |
| *IA,i* | 6 | 7±0 | 11.5±0.71 | 16±0 | 23.5±0.71 | 28±1.41 | 32±1.41 | 40±0 |
| *IA,i* | 7 | 5.5±0.71 | 12±1.41 | 19.5±3.54 | 27±4.24 | 30±4.24 | 33.5±3.54 | 35.5±2.12 |
| *IA,i* | 8 | 9±0 | 14.5±0.71 | 21.5±0.71 | 30.5±0.71 | 34±0 | 37.5±0.71 | 40±0 |
| *IA,i* | 9 | 8.5±0.71 | 13.5±0.71 | 20±0 | 26.5±2.12 | 30.5±2.12 | 34.5±2.12 | 40±0 |
| *IA,i* | 10 | 5±0 | 10.5±0.71 | 19.5±0.71 | 26±0 | 29.5±0.71 | 33.5±0.71 | 40±0 |

**Table S2.** Expression levels of genes of *IA,a* compared to the parent *A,a* (control). The hypothesis test performed by REST2009 and based on 1000 iterations provides a *p-value* which represents the probability of the alternate hypothesis that the difference between *IA,a* and *A,a* is due only to chance.

| Gene ID | Fold change (expression level) | *p-value* |
|---|---|---|
| **46054** | 3.64 | 0.05 |
| **59167** | 0.27 | 0.00 |
| **147699** | 1.17 | 0.52 |
| **143314** | 2.75 | 0.00 |
| **33584** | 89.06 | 0.00 |
| **109183** | 0.29 | 0.00 |
| **148283** | 32.87 | 0.00 |
| **108968** | 0.22 | 0.00 |
| **46250** | 0.62 | 0.30 |

**Table S3.** Expression levels of genes of *IA,i* compared to the parent *I,i* (control). The hypothesis test performed by REST2009 and based on 1000 iterations provides a *p-value* which represents the probability of the alternate hypothesis that the difference between *IA,i* and *I,i* is due only to chance.

| Gene ID | Fold change (expression level) | *p-value* |
|---|---|---|
| **46054** | 1.44 | 0.22 |
| **59167** | 1.70 | 0.17 |
| **147699** | 0.28 | 0.00 |
| **143314** | 0.96 | 0.93 |
| **33584** | 0.27 | 0.06 |
| **109183** | 0.59 | 0.23 |
| **148283** | 0.02 | 0.00 |
| **108968** | 0.57 | 0.08 |
| **46250** | 0.82 | 0.58 |

**Table S4.** Expression levels of genes of hybrid *IA,i* compared to the hybrid *IA,a*. The hypothesis test performed by REST2009 and based on 1000 iterations provides a *p-value* which represents the probability of the alternate hypothesis that the difference between *IA,i* and *IA,a* is due only to chance.

| Gene ID | Fold change (expression level) | *p-value* |
|---|---|---|
| **46054** | 2.335 | 0.01 |
| **59167** | 1.330 | 0.49 |
| **147699** | 0.959 | 0.92 |
| **143314** | 1.404 | 0.41 |
| **33584** | 2.170 | 0.01 |
| **109183** | 0.815 | 0.63 |
| **148283** | 4.993 | 0.01 |
| **108968** | 0.720 | 0.41 |

**Table S5**. List of isolates used in the work and their Mycotheca Universitatis Taurinensis (MUT) accession numbers.

| Genotype | Species | MUT accession N. |
|---|---|---|
| ***I,i*** | *H. irregulare* | MUT00005666 |
| ***A,a*** | *H. annosum* s.s. | MUT00003656 |
| ***IA,i*** | Artificial heterokaryotic hybrid (with *H. irregulare* mitochondria) | MUT00005668 |
| ***IA,a*** | Artificial heterokaryotic hybrid (with *H. annosum* s.s mitochondria) | MUT00005669 |

**Table S6.** List of the *Heterobasidion* spp. primers used in the current work and relative Temperature of annealing (Ta).

| Sequence | Name | Ta (°C) |
|---|---|---|
| GGCAGTTTGGTGCACTTAC | *Het_Trypt_metF* | ***60*** |
| CCACCAGCCTGCGTACTT | *Het_Trypt_metR* | |
| AGTGCTGCAACTCTGTCCAG | *Het46054f* | ***60*** |
| CAATTGATGCCAACCATGGTG | *Het46054r* | |
| AAACGGGACGCTGGTTGAT | *Het108968Af* | ***60*** |
| GTGGGCAAAGGCGAAGTAT | *Het108968Ar* | |
| CGGAGCCTATCACGACTGA | *Het436250Af* | ***60*** |
| AGTGCCGGAGACCTGAGAT | *Het436250Ar* | |
| GATGTCGTGAAGCGGGAAGG | *Het109183Af* | ***60*** |
| TATTTCGCCAGATCGCTCGC | *Het109183Ar* | |
| GACGGCGGCATTGTGTTCTA | *Het148283Af* | ***60*** |
| GACTTCAACCAGCTCCTGCC | *Het148283Ar* | |
| GCGCCAAGACTCATCCTAGC | *Het143314Af* | ***60*** |
| TCATAGTGCGACTCCTCGCT | *Het143314Ar* | |
| AACTCCGTGATTTTCGGCCC | *Het147699Af* | ***62*** |
| ATCAGGGTGCCGATCCAGAA | *Het147699Ar* | |
| ATTCTACTCACGCCGACCGA | *Het33584Af* | ***60*** |
| CGAAGGCCGAGCACAACATA | *Het33584Ar* | |
| ATGTCGTAGAGGAGGGCGTC | *Het59167Af* | ***62*** |
| TTCACTTGCCGTGTACGGTG | *Het59167Ar* | |

**Table S7**. Average Ct values of the nine selected genes in the four different genotypes (Ct values of each of the three biological replicates ± SD of technical replicates).

| | | | | | | | | | | | | | |
|---|---|---|---|---|---|---|---|---|---|---|---|---|---|
| | ***Biological replicate*** | ***Aa1*** | ***Aa2*** | ***Aa3*** | ***Ii1*** | ***Ii2*** | ***Ii3*** | ***IA,i1*** | ***IA,i2*** | ***IA,i3*** | ***IA,a1*** | ***IA,a2*** | ***IA,a3*** |
| ***Gene ID*** | | | | | | | | | | | | | |
| ***Trypt_met*** | | 19.51 | 18.56 | 23.35 | 22.77 | 22.70 | 18.11 | 24.89 | 24.17 | 23.21 | 21.26 | 20.62 | 22.50 |
| ***SD*** | | 0.32 | 0.27 | 0.66 | 0.24 | 0.28 | 0.32 | 0.84 | 0.46 | 0.21 | 0.26 | 0.43 | 0.47 |
| ***46054*** | | 15.27 | 14.58 | 23.90 | 20.42 | 19.41 | 15.74 | 21.63 | 21.51 | 19.54 | 17.38 | 16.27 | 17.47 |
| ***SD*** | | 0.25 | 0.37 | 0.63 | 0.24 | 0.12 | 0.19 | 0.21 | 0.21 | 0.12 | 0.16 | 0.37 | 0.15 |
| ***59167mit*** | | 16.63 | 16.24 | 21.21 | 21.95 | 21.85 | 18.69 | 25.13 | 24.34 | 22.43 | 20.50 | 19.65 | 22.63 |
| ***SD*** | | 0.01 | 0.09 | 0.10 | 0.81 | 0.25 | 0.08 | 0.96 | 0.44 | 0.79 | 0.05 | 0.33 | 0.67 |
| ***147699*** | | 16.45 | 14.75 | 20.09 | 17.32 | 16.44 | 13.39 | 20.26 | 20.75 | 20.27 | 17.32 | 16.90 | 19.35 |
| ***SD*** | | 0.86 | 0.25 | 0.38 | 0.32 | 0.28 | 0.30 | 0.15 | 0.07 | 0.12 | 0.28 | 0.18 | 0.11 |
| ***143314*** | | 18.23 | 18.77 | 19.66 | 17.89 | 17.62 | 19.22 | 20.10 | 21.75 | 21.77 | 19.13 | 19.95 | 19.18 |
| ***SD*** | | 0.07 | 0.07 | 0.08 | 0.88 | 0.16 | 0.15 | 0.04 | 0.10 | 0.07 | 0.08 | 0.06 | 0.11 |
| ***33584mit*** | | 20.32 | 18.65 | 23.48 | 22.71 | 22.68 | 20.41 | 25.64 | 25.28 | 24.31 | 19.83 | 19.72 | 20.46 |
| ***SD*** | | 0.69 | 0.50 | 0.82 | 0.27 | 0.44 | 0.07 | 0.27 | 0.10 | 0.17 | 0.13 | 0.22 | 0.03 |
| ***109183*** | | 17.38 | 16.73 | 22.55 | 21.61 | 20.45 | 18.95 | 23.71 | 24.83 | 23.44 | 21.25 | 20.51 | 23.22 |
| ***SD*** | | 0.16 | 0.20 | 0.03 | 0.35 | 0.40 | 0.01 | 0.29 | 0.24 | 0.45 | 0.26 | 0.17 | 0.24 |
| ***148283*** | | 25.45 | 23.93 | 26.84 | 20.35 | 17.65 | 15.38 | 25.60 | 24.29 | 25.03 | 23.53 | 22.35 | 23.19 |
| ***SD*** | | 0.67 | 0.32 | 0.20 | 0.05 | 0.12 | 0.24 | 0.18 | 2.03 | 0.70 | 0.29 | 0.31 | 0.23 |
| ***108968*** | | 19.29 | 18.40 | 21.56 | 22.52 | 22.23 | 19.49 | 25.39 | 25.58 | 24.37 | 21.11 | 23.37 | 24.39 |
| ***SD*** | | 0.22 | 0.08 | 0.36 | 0.37 | 0.27 | 0.38 | 0.92 | 0.68 | 0.29 | 0.13 | 0.34 | 0.23 |
| ***46250*** | | 21.14 | 20.43 | 25.53 | 24.54 | 23.61 | 19.66 | 26.34 | 25.20 | 26.22 | 21.71 | 24.21 | 26.22 |
| ***SD*** | | 1.50 | 1.74 | 1.00 | 0.06 | 0.82 | 0.47 | 0.58 | 0.10 | 0.40 | 0.15 | 0.93 | 0.40 |
